# Synaptotagmin-like protein 1 is a potential diagnostic and prognostic biomarker in endometrial cancer based on bioinformatics and experiments

**DOI:** 10.1186/s13048-023-01097-2

**Published:** 2023-01-19

**Authors:** Cai Meijuan, Xu Meng, Liu Fang, Wang Qian

**Affiliations:** 1 Department of Clinical Laboratory, Qilu Hospital of Shandong University, No.107 Wenhua West Road, 250013 Jinan, Shandong China; 2 Department of Clinical Laboratory, Qilu Hospital of Shandong University (Qingdao), Qingdao, Shandong China; 3 Department of Pathology, Qingdao Chengyang People’s Hospital, No.758 Hefei Road, Shandong 266035 Qingdao, China

**Keywords:** Endometrial cancer, Synaptotagmin-like protein 1, Prognosis, DNA methylation, Immune infiltration

## Abstract

Endometrial cancer (EC) is one of the most common gynecologic malignancies. Identification of potential EC biomarkers is essential to improve the prognosis and development of therapies against EC. Synaptotagmin-like protein 1 (SYTL1), as a small GTPase Rab27 effector, mainly plays a role in vesicle trafficking and cytotoxic granule exocytosis in lymphocytes. However the role of SYTL1 in EC remains uncertain. We performed a comprehensive assessment of the relationship between SYTL1 and patient diagnosis and prognosis by analysis of EC patients’ data from TCGA. We employed the LinkedOmics and Search Tool for the Retrieval of Interacting Genes/Proteins (STRING) database to analyze the biological function of SYTL1 in EC. In addition, the correlation between SYTL1 expression and its DNA methylation was performed by using cBioportal, UALCAN, TCGA Wanderer and MethSurv databases. We further assessed the link between SYTL1 and tumor-infiltrating immune cells by using gene set variation analysis (GSVA).

**Results** We found that SYTL1 was highly expressed in EC patients and cell lines. And increased expression of SYTL1 was associated with age, clinical stage, histological type, histological grade and good overall survival (OS).SYTL1 DNA methylation is negatively associated with SYTL1 expression and UCEC patients’ OS. SYTL1 expression is closely correlated with immune infiltration. Furthermore, we carried out in vitro experiments to verify the results of bioinformatic analysis.

**Conclusion** Our results demonstrated that the elevation of SYTL1 expression is associated with good OS and SYTL1 might be a potential diagnostic and prognostic marker in EC.

**Supplementary Information:**

The online version contains supplementary material available at 10.1186/s13048-023-01097-2.

## Background

Endometrial cancer (EC) is the fourth most common malignancy in women, which accounts for more than 76,000 deaths among women each year worldwide [1]. According to the latest global cancer data from the World Health Organization in 2020, EC is one of the top 10 new cancer cases, with more than 80,000 new patients diagnosed in China. Five-year overall survival (OS) rates for EC vary according to the stage at diagnosis. Most EC patients are diagnosed in early stage and have a good prognosis with 5-year OS rates of nearly 90% [2]. Unfortunately, roughly 30% of individuals diagnosed with advanced stage (stage III or IV) have a poor prognosis, with a worse 5-year survival rate of 60% and 20%, respectively [3].Thus, great efforts are needed to identify new clinically feasible molecular biomarkers for EC diagnosis and then to improve the outcome of EC.

Synaptotagmin-like protein 1 (SYTL1, also named JFC1, SLP1), is a member of the synaptotagmin-like protein family of secretory factors characterized with a Rab-binding domain at N-terminal and two tandem-C2 domains at C-terminal [4]. SYTL1 differentially regulates the secretion of prostate-specific antigen and prostatic-specific acid phosphatase [5]. SYTL1 is involved in controlling Rab8 membrane dynamics by binding specifically to Rab8 [6]. In granulocytes, SYTL binding to Rab27A constitutes key components of the secretory machinery of azurophilic granules [7] and is involved in amylase secretion [8]. In platelets, SYTL1 interacts with GTPase-activating protein Rap1GAP2 to regulate dense granule secretion [9]. During exocytosis, SYLT1 interacting with the RhoA-GTPase-activating protein Gem-interaction protein regulates vesicular trafficking through cortical actin [10]. Previous study was mainly focused on the function of SYTL1 about the regulation of secretion and exocytosis. However the role of SYTL1 in tumor progression remains unclear. In prostate cancer cell lines, SYTL1 is transcriptionally activated by nuclear factor-κB and up-regulated by tumor necrosis factor α[11]. It is reported that SYTL1 binds to the plasma membrane via interacting with phosphatidylinositol 3,4,5-trisphosphate (PIP3) with ATPase capacity [12].The phosphoinositide 3-kinase and its product PIP3 play a central role incellular physiology and mediate critical cellular processes, such as cell proliferation, survival and cytoskeletal reorganization during tumor development[13].We guess that SYTL1 might be involved in cancer progression. Thus, the objective of the current study is to evaluate the diagnostic and prognostic value of SYTL1 expression in human EC through bioinformatics analysis and experiments.

We evaluated the diagnostic and prognostic value of SYTL1 expression in UCEC by analyzing the patients’ data from TCGA. We employed the LinkedOmics and STRING database to analyze the biological function of SYTL1. In addition, the correlation between SYTL1 expression and the methylation levels was performed by using cBioportal, UALCAN, TCGA Wanderer and MethSurv databases. We further assessed the link between SYTL1 and tumor-infiltrating immune cells by single sample GSEA method from R package GSVA. Furthermore, we carried out in vitro experiments to verify the results of bioinformatics analyisis. Our results demonstrated that SYTL1 might be a potential diagnostic and prognostic marker in EC.

## Results

### SYTL1 is highly expressed in CESC, OV and UCEC

**Fig. 1:**
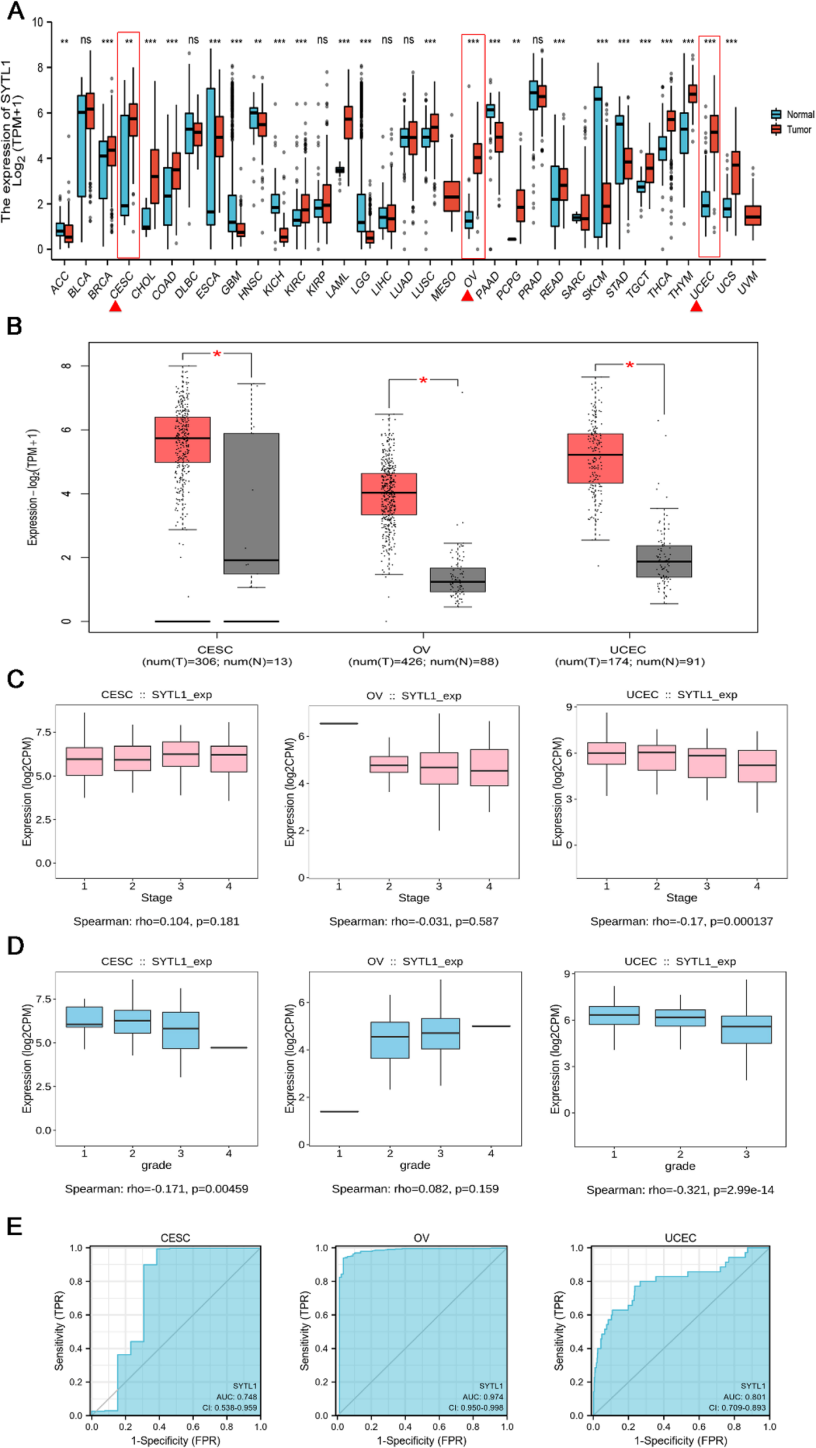
A. SYTL1 expression status in 33 different cancer tissues compared to the normal tissues from TCGA database. B. SYTL1 expression levels in CESC, OV and UCEC compared with normal tissues in GEPIA database. C and D. The association between SYTL1 and clinical stageand histologic grade in CESC, OV and UCEC analyzed in TISIDB. E. The ROC of diagnosis to distinguish tumor from normal tissues. ***p < 0.001, **p < 0.01, *p < 0.05

To explore the possible role of SYTL1, we analyzed its expression in 33 types of different cancers. In Fig. 1 A, SYTL1 was significantly upregulated in malignant tumors of female reproductive system, including CESC, OV and UCEC. The similar results were observed in GEPIA2 online database (Fig. 1B).These data suggest that SYTL1 is upregulated in CESC, OV and UCEC.

### Value of high SYTL1 expression in diagnosis and predicting prognosis in UCEC

**Fig. 2:**
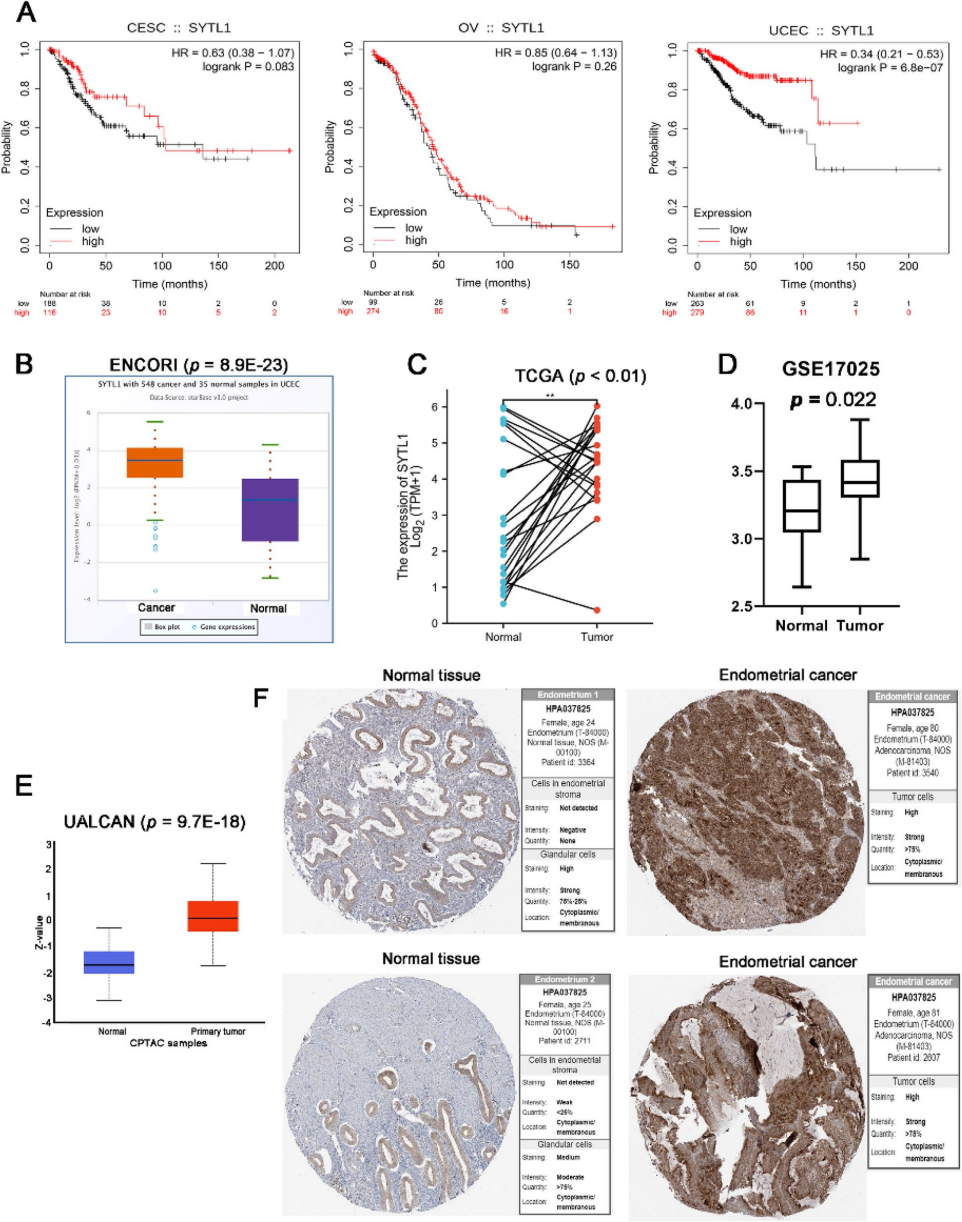
A. KM curves for OS of CESC, OV and UCEC patients according to SYTL1 expression levels. B. The mRNA expression of SYTL1 in UCEC tissues compared with normal tissues analyzed by using ENCORI database. C. SYTL1 expression in EC tissues compared with the corresponding adjacent normal tissues from TCGA data. D. the mRNA levels of SYTL1 in GSE17025. E. The protein expression of SYTL1 in EC and normal tissues analyzed by UALCAN database. F. Immunohistochemistry staining for SYTL1 in human endometrium and EC tissues were downloaded from HPA database. ***p < 0.001, **p < 0.01, *p < 0.05

As shown in Fig. 1 C and 1D, the expression of SYTL1 remarkably negatively correlated with clinical stage in UCEC (r = -0.17, *p* = 0.000137), histologic grade in CESC (r = -0.171, *p* = 0.00459) and UCEC (r = -0.321, *p* = 2.99e-14).ROC curve was performed to further explore the diagnostic value of SYTL1 in CESC (AUC = 0.748), OV (AUC = 0.974) and UCEC (AUC = 0.801) in Fig. 1E. According to the KM survival curves in Fig. 2 A, higher SYTL1 expression showed a better OS in UCEC (*p* = 6.8e-07), however no significant correlation between SYTL1 expression and OS was observed in CESC (*p* = 0.083) and OV (*p* = 0.26). Based on the above results, we further investigated the role of SYTL1 in UCEC. In ENCORI database[14],the mRNA level of SYTL1 in UCEC tissues was higher than that in the normal samples (*p* = 8.9e-23, Fig. 2B). Meanwhile, the transcription level of SYTL1 in 23 EC tissues was elevated than that in the matched adjacent normal tissuesbased on TCGA database (*p* < 0.01, Fig. 2 C). By analyzing GEO data GSE17025, we also found that SYTL1 was highly expressed in EC (Fig. 2D, *p* = 0.022). We further identified the differentially expression of SYTL1 protein using the CPTAC dataset in UALCAN portal. In Fig. 2E, SYTL1 protein level in the primary UCEC tissues was significantly higher than that in normal tissues (*p* = 9.7e-18).We further extracted the IHC images from HPA database [15], and found that the signals of SYTL1 in UCEC tissues were higher than those in endometrial stroma and glandular cells (Fig. 2 F).

### SYTL1 expression is correlated with clinicopathological features of UCEC

To investigate the relationship between SYTL1 expression and clinicopathological characteristic in UCEC, we obtained a total of 552 EC samples and 35 adjacent EC tissues with both clinical and gene expression from TCGA database (Table 1). In our study cohort, Stage I disease was found in 342 patients (62.1%), Stage II in 51 (9.2%), Stage III in 130 (23.5%) and Stage IV in 29 (5.2%). Most tumor (*n* = 410, 74.3%) were of endometrioid adenocarcinoma, 4.3% (*n* = 24) were mixed serous and endometrioid adenocarcinoma and 21.4% (*n* = 118) were serous endometrioid adenocarcinoma. Histological grade G1 was found in 98 patients (18.2%), G2 in 120 (22.1%) and G3 in 323 (59.7%).

**Table 1:** SYTL1 expression associated with clinicopathologic characteristics (logistic regression)

Characteristics	Total(N)	Odds Ratio(OR)	*P* value
Age (> 60 vs. <=60)	549	0.603 (0.425–0.853)	0.004**
Weight (> 80 vs. <=80)	528	1.272 (0.903–1.794)	0.169
Clinical stage (Stage II&Stage III&Stage IV vs. Stage I)	552	0.610 (0.430–0.861)	0.005**
Histological type (Mixed&Serous vs. Endometrioid)	552	0.208 (0.133–0.319)	< 0.001***
Histologic grade (G2&G3 vs. G1)	541	0.352 (0.217–0.560)	< 0.001***
Tumor invasion(%) ( > = 50 vs. <50)	474	0.866 (0.603–1.244)	0.436

*p < 0.05, **p < 0.01, ***p < 0.001

**Fig. 3:**
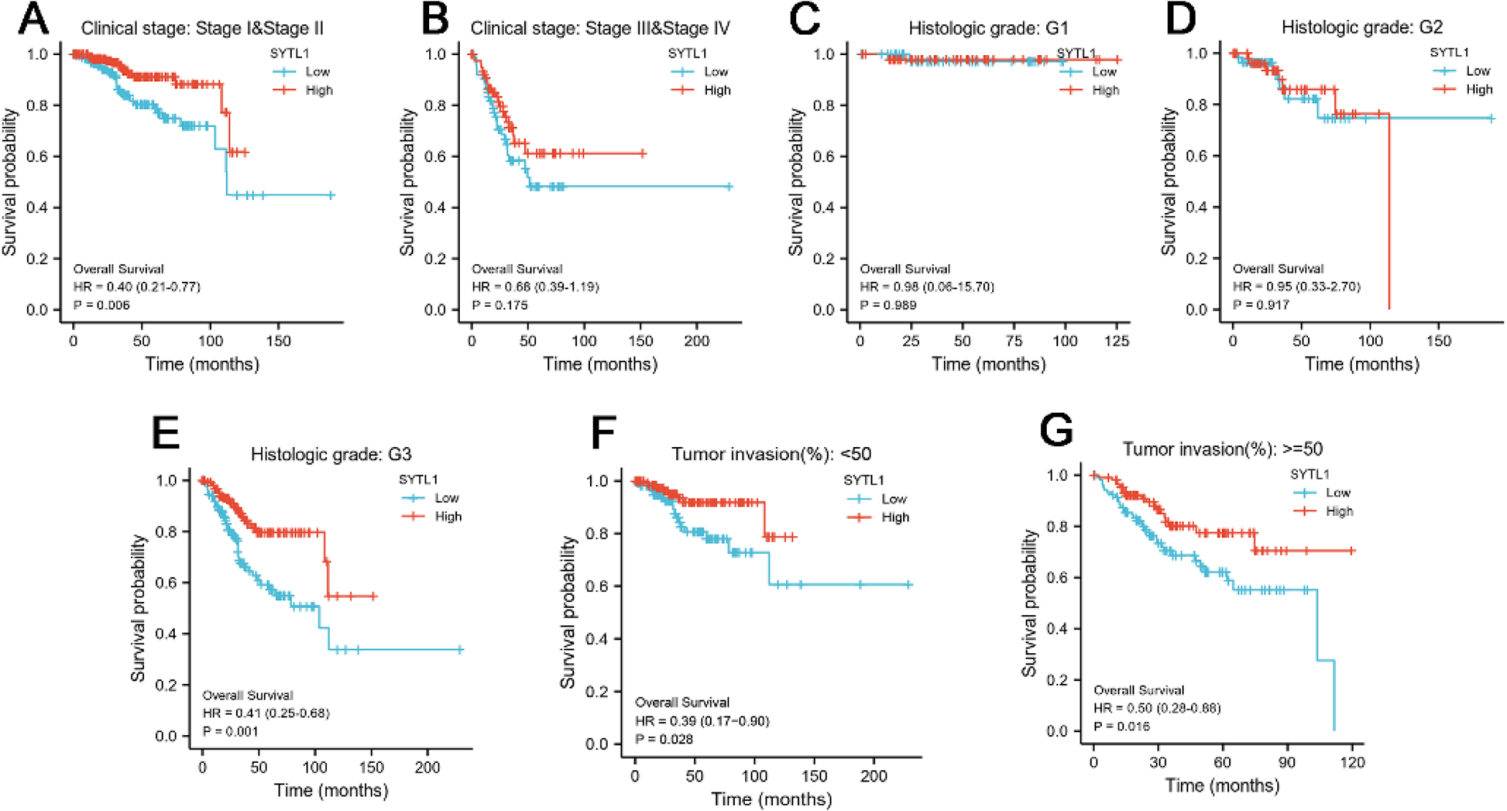
KM curve for SYTL1 in all tumor patients. A-G, subgroup analysis for Stage I&Stage II, Stage III&Stage IV, G1, G2, G3, tumor invasion < 50% and tumor invasion > = 50%

To explore the prognostic value of SYTL1 expression in EC, we used R package to perform KM survival subgroup analysis by different clinical features (Fig. 3). The results demonstrated that high SYTL1 expression was obviously associated with good prognosis in EC patients Stage I&Stage II (*p* = 0.002), Histologic grade G3 (*p* = 0.003), Tumor invasion < 50% (*p* = 0.023) and Tumor invasion > = 50% (*p* = 0.002), and the expression of SYTL1 was not significantly related with prognosis in EC patients Stage III&Stage IV (*p* = 0.411), histologic grade G1 (*p* = 0.956), and histologic grade G2 (*p* = 0.397). In Table 2, the univariate analysis using logistic regression revealed that SYTL1-high was significantly correlated with good OS (HR = 0.337, *p* < 0.001). Upon further multivariate analysis, SYTL1 expression remained independently correlated with OS and clinical stage in TCGA datasets. These data show that SYTL1 may be an independent prognosis indictor for inducing cancer risk and extending patients’ OS.

**Table 2:** Univariate and multivariate Cox regression analysis of clinical characteristics associated with overall survival

Characteristics	Total(*N*)	Univariate analysis		Multivariate analysis	
		Hazard ratio (95% CI)	*P* value	Hazard ratio (95% CI)	*P* value
Age (> 60 vs. < = 60)	549	1.847 (1.160–2.940)	0.010*	1.567 (0.959–2.562)	0.073
Weight (> 80 vs. < = 80)	527	1.060 (0.699–1.607)	0.784		
Clinical stage (Stage II&Stage III&Stage IV vs. Stage I)	551	3.270 (2.145–4.984)	< 0.001***	2.775 (1.782–4.320)	< 0.001***
Histological type (Mixed&Serous vs. Endometrioid)	551	2.628 (1.746–3.957)	< 0.001***	1.312 (0.825–2.087)	0.251
SYTL1 (High vs. Low)	551	0.337 (0.215–0.527)	< 0.001***	0.446 (0.278–0.715)	< 0.001***

^*^p < 0.05, **p < 0.01, ***p < 0.001

### Functional analysis of SYTL1-related genes

**Fig. 4:**
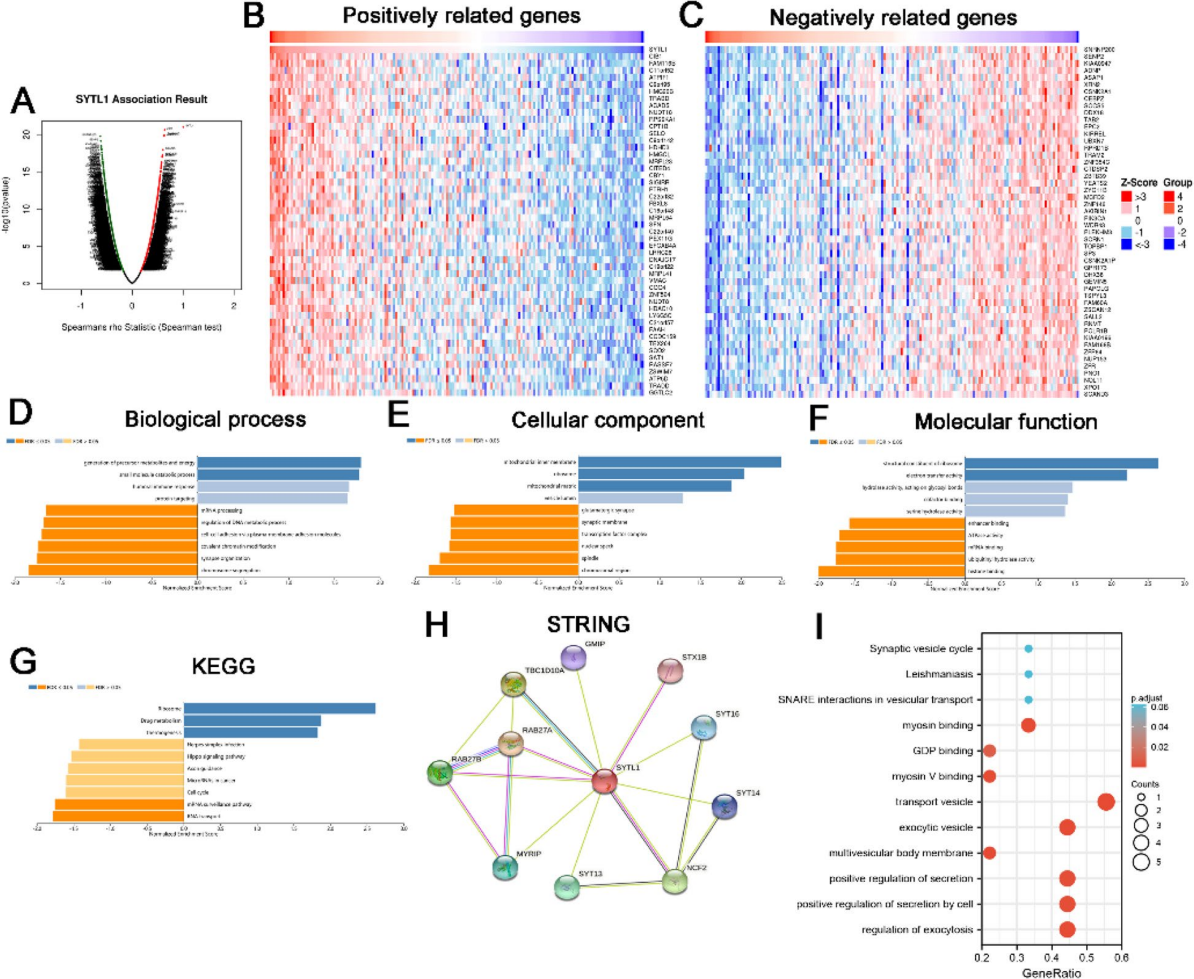
**A**. The significantly associated genes with SYTL1 distinguished by Pearson test in EC cohort from the LinkedOmics database. **B** and **C**. Top 50 genes positively and negatively related to SYTL1 in EC showed by heatmaps. Red presents positively linked genes and blue represents negatively linked genes. E-H. GO annotations (GO-Biological process (**D**), GO-Cellular Component (**E**), GO-Molecular Function (**F**)) and KEGG pathways (**G**) of SYTL1 in EC cohort. **H**. Protein-protein interaction network obtained from STRING. **J**. GO term and KEGG enrichment analysis of 10 functional partner genes of SYTL1.

To further explore the biological function of SYTL1 in EC, the LinkFinder module in the LinkedOmics web portal was employed to obtain the differentially expressed genes related to SYTL1 in TCGA-UCEC. As shown in Fig. 4 A, it showed that 1,586 genes (marked in dark red dots) positively associated with SYTL1, and 3,136 genes (dark green dots) negatively correlated (false discovery rate [FDR] < 0.001) based on the Spearman test. The top 50 positively and top 50 negatively correlated genes were displayed by heat maps in Fig. 4B C.

GO term annotation (Fig. 4D F) showed that the differentially expressed genes related with SYTL1 were localized in mitochondrial inner membrane, ribosome, mitochondrial matrix and vesicle lumen, where they primarily participated in generation of precursor metabolites and energy, small molecule catabolic process, humoral immune response, protein targeting, chromosome segregation, and mRNA processing, etc. They acted as structural constituent of ribosome, electron transfer activity, and histone binding. The functions of these DEGs are mainly enriched in Ribosome, RNA transport, mRNA surveillance pathway, drug metabolism, cell cycle and Thermogenesis through the KEGG pathway analysis (Fig. 4G).

We further conducted a network analysis of SYTL1 by using STRING. In Fig. 4H, the top ten functional partner genes were selected with a high degree of connectivity, including RAB27A, TBC1D10A, NCF2, RAB27B, SYT13, MYRIP, SYT16, SYT14, GMIP and STX1B (Table 3). In combination with enrichment analysis via GO/KEGG, we found that RAB27A, RAB27B, MYRIP, SYT13 and STX1B are enriched in the transport vesicle shown in Fig. 4I.

**Table 3:** The detailed information of SYTL1-related genes

Gene Symbol	Annotation	Score
RAB27A	Ras-related protein Rab-27 A	0.998
TBC1D10A	TBC1 domain family member 10 A	0.843
NCF2	Neutrophil cytosol factor 2	0.782
RAB27B	Ras-related protein Rab-27B	0.764
SYT13	Synaptotagmin-13	0.74
MYRIP	Myosin viia and rab interacting protein	0.735
SYT16	Synaptotagmin-16	0.731
SYT14	Synaptotagmin-14	0.73
GMIP	GEM-interacting protein	0.659
STX1B	Syntaxin-1B	0.654

### Protein post-translational modification and genetic alternation analysis

We employed ProsphoNET database (https://www.phosphosite.org/homeAction) to analyze the putative phosphorylation sites. In Fig S1A, four SYTL1 phosphorylation sites (H120, S216, Y304 and S392) were experimentally supported by at least 5 references. In UALCAN portal, the differences in SYTL1 phosphorylation levels between primary tumor and normal tissues were analyzed. By using CPTAC dataset, the S216 locus exhibits no significant difference between tumor and normal tissues (Fig S1B, *p* = 0.36), however the S392 locus exhibits a higher phosphorylation level in primary tumor tissues compared with normal tissues (Fig S1C, *p* = 4.43E-07). These data indicate that more experiments need to be carried out for the exploration of the potential role of S392 phosphorylation in EC tumorigenesis.

The identification of genetic aberrations involved in the pathogenesis of EC is leading to the development of new therapeutic options for immunotherapy and targeted therapy. We investigated the genetic alternation status of SYTL1 in different tumor samples according to the cBioPortal database. Respective results revealed that SYTL1 was altered in 140 of the 10,953 patients included on the TCGA (1%). The highest alteration ratio was related to mutations (Fig S2A). Fig S2B showed the sites of the SYTL1 genetic alteration in UCEC. Additionally, we explored the potential association between genetic alternation of SYTL1 and clinical survival prognosis of cases. In Fig S2C-S2F, no significant difference about prognosis in overall (*p* = 0.540), disease-free (*p* = 0.477), progression-free (*p* = 0.561) and disease-specific (*p* = 0.873) survival was observed between cases with altered SYTL1 and without SYTL1 alternation.

### SYTL1 DNA methylation is associated with UCEC patients survival

**Fig. 5:**
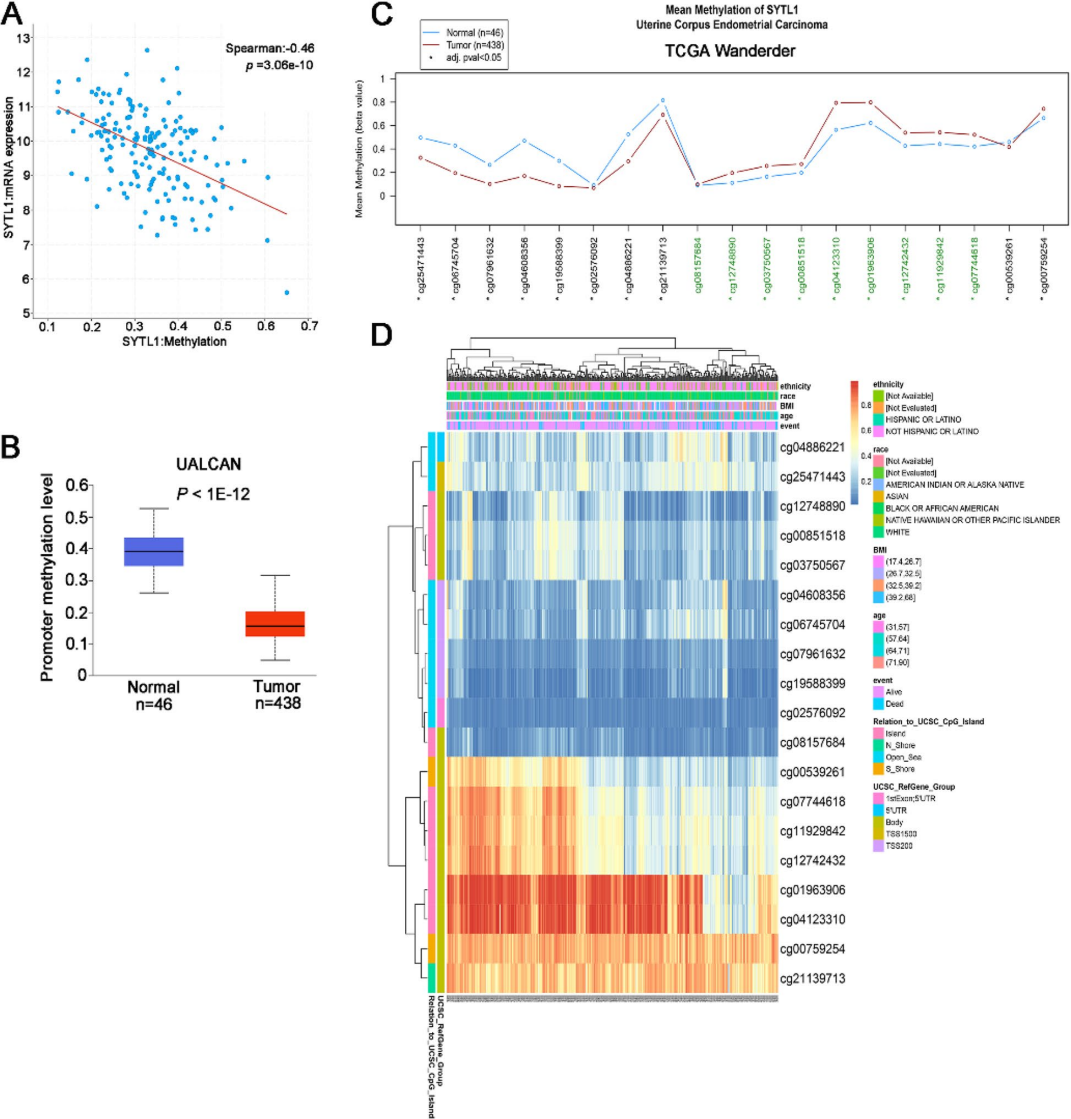
**A**. The correlation between SYTL1 methylation and its expression level analyzed by cBioportal database. **B**. The promoter methylation level of SYTL1 in EC tissues and normal tissues by UALCAN analysis. **C**. Mean methylation of SYTL1 in EC in TCGA Wanderer. **D**. Visualization between the methylation level of SYTL1 and its expression

According to the cBioPortal database, we found that SYTL1 expression was negatively correlated with SYTL1 methylation level (Fig. 5 A, r = -0.460, *p* = 3.06e-10).By using UALCAN portal, we found that promoter methylation level of SYTL1 in normal patients was significantly higher than that in tumor patients (Fig. 5B, p = 1e-12). TCGA Wanderer web tool was employed to analyze the methylation pattern of SYTL1 in promoter region of normal vs. UCEC specimens. As shown in Fig. 5 C, there were 19 probes in the region (chr1:27,667,000–27,681,000), including 9 probes located in CpG island (marked in green). Eighteen of 19 probes in 450 methylation array exhibited significant difference between normal and UCEC specimens.

We further investigated the DNA methylation levels of SYTL1 with the prognostic values of each single CpG by using the MethSurv tool. In Fig. 5D, cg01963906 and cg04123310 had the highest DNA methylation. The methyaltion level of nine CpG sites significantly correlated with prognosis, including cg00539261, cg00851518, cg03750567, cg04608356, cg06745704, cg07744618, cg07961632, cg12742432 and cg12748890 (*p* < 0.05, Table 4). These data show that DNA methylation is negatively related with SYTL1 expression and UCEC patients’ OS.

**Table 4:** Effect of hypermethylation level on prognosis in UCEC

CpG	HR	95%CI	*P* value
**Body_S_Shore_cg00539261**	**0.509**	**0.308–0.841**	0.008**
Body_S_Shore_cg00759254	1.425	0.868–2.339	0.161
**Body_Island_cg00851518**	**0.549**	**0.338–0.893**	0.016*
Body_Island_cg01963906	0.658	0.401–1.081	0.099
1stExon;5'UTR_Open_Sea_cg02576092	1.68	0.92–3.066	0.091
**Body_Island_cg03750567**	**0.471**	**0.291–0.763**	0.002**
Body_Island_cg04123310	0.63	0.351–1.132	0.122
**TSS200_Open_Sea_cg04608356**	**1.962**	**1.055–3.647**	0.033*
5'UTR_Open_Sea_cg04886221	1.509	0.934–2.438	0.122
**TSS200_Open_Sea_cg06745704**	**2.339**	**1.164–4.702**	0.017*
**Body_Island_cg07744618**	**0.624**	**0.39–0.998**	0.049*
**TSS200_Open_Sea_cg07961632**	**2.151**	**1.32–3.507**	0.002**
Body_Island_cg08157684	0.803	0.484–1.334	0.397
Body_Island_cg11929842	0.605	0.325–1.124	0.112
**Body_Island_cg12742432**	**0.541**	**0.335–0.875**	0.012*
**Body_Island_cg12748890**	**0.465**	**0.291–0.743**	0.001**
TSS200_Open_Sea_cg19588399	1.554	0.969–2.492	0.067
Body_N_Shore_cg21139713	1.256	0.756–2.087	0.380
TSS1500_Open_Sea_cg25471443	1.698	0.931–3.097	0.084

^*^p < 0.05, **p < 0.01, ***p < 0.001

### SYTL1 expression is associated with immune infiltration

**Fig. 6:**
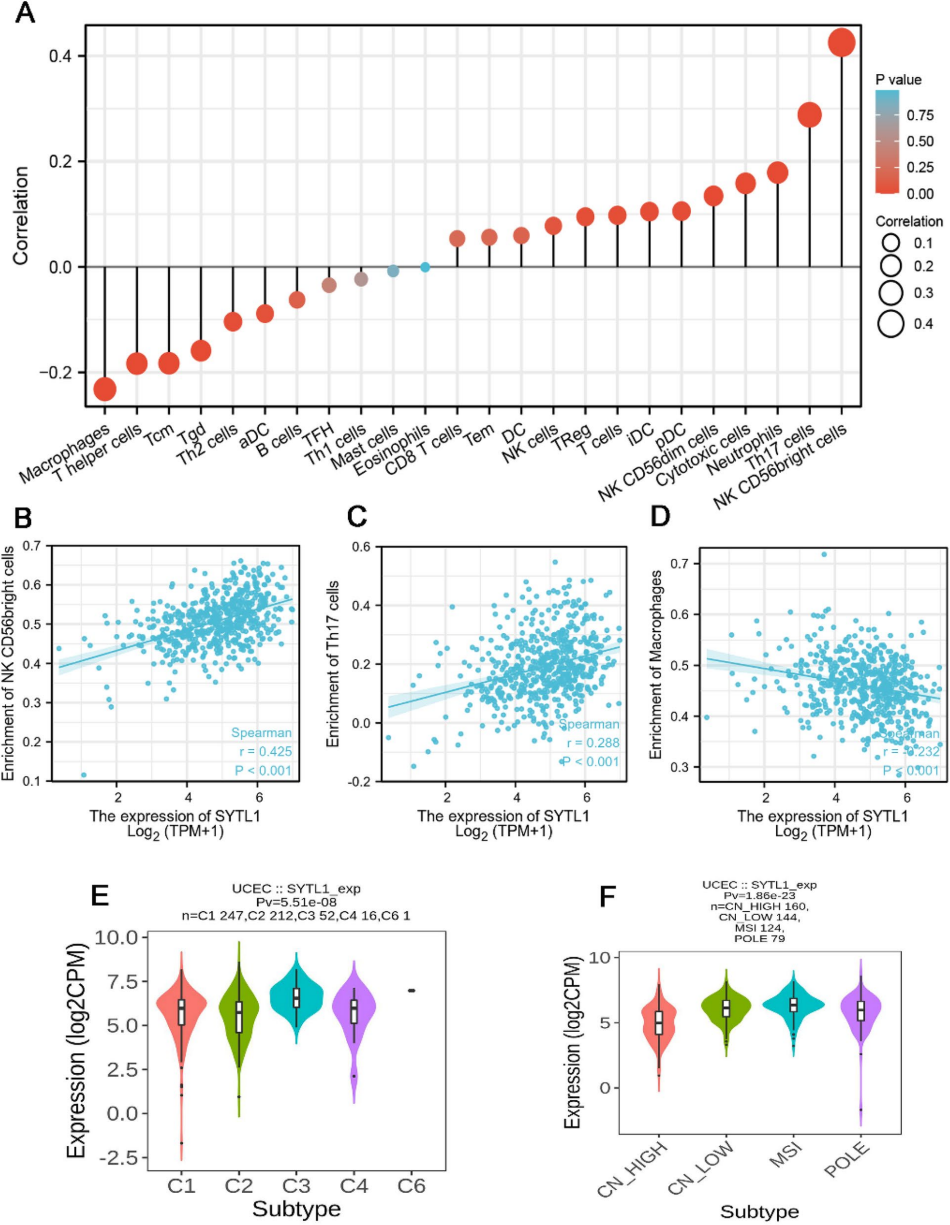
**A**. The association between SYTL1 expression and immune cell infiltration. **B**-**C**. A positive relationship between SYTL1 expression and NKCD56bright cell (B) or Th17 (C). **D**. A negative correlation between SYTL1 expression and Macrophages. E-F. Correlation between SYTL1 expression and molecular subtypes (E) or immune subtypes (F).aDC, activated DC; iDC, immature DC; pDC, plasmacytoid DC; Tcm, T central memory; Tem, T effector memory; Tfh, T follicular helper; Tgd, T gamma delta

We further analyzed the correlation between SYTL1expression and immune cell infiltration by ssGSEA with Spearman r. In Fig. 6 A, the number of NK CD56bright cells, Th17, Neutrophils, Cytotoxic cells, NK CD56dim cells, pDC, iDC, T cells and Treg cells were positively correlated with SYTL1 expression. The strongest positive correlation was observed between the number of NK CD56bright cells, Th17 and SYTL1 expression. However, SYTL1 expression was negatively correlated with the number of Macrophages, T helper, Tcm, Tgd, Th2 and activated dendritic cells (aDCs)( *p* < 0.05). The strongest negative correlation was observed between the number of Macrophages and SYTL1 expression.

EC was classified into five kinds of immune subtype, including wound healing (C1), IFN-gamma dominant (C2), inflammatory (C3), lymphocyte depleted (C4), immunologically quiet (C5), and TGF-β dominant (C6). TCGA research network identified four prognostic molecular subgroups of EC: copy number high (CN_HIGH), copy number low (CN_LOW), microsatelliteinstability (MSI) and DNA polymerase-epsilon (POLE). TISIDB was applied to explore the correlation between SYTL1 level and immune and molecular subtype. In Fig. 6E, SYTL1 expression was significantly related with C1, C2, C3, C4, C5, and C6 subtypes (*p* = 5.51e-08). In addition, we also found that SYTL1 expression was markedly correlated with CN_HIGH, CN_LOW, MSI and POLE subgroups (*p* = 1.86e-23)in Fig. 6 F. These results indicated the relationship between SYTL1 expression and immune subtypes and molecular subgroups.

### SYTL1 is involved in cell proliferation and invasion in EC

**Fig. 7:**
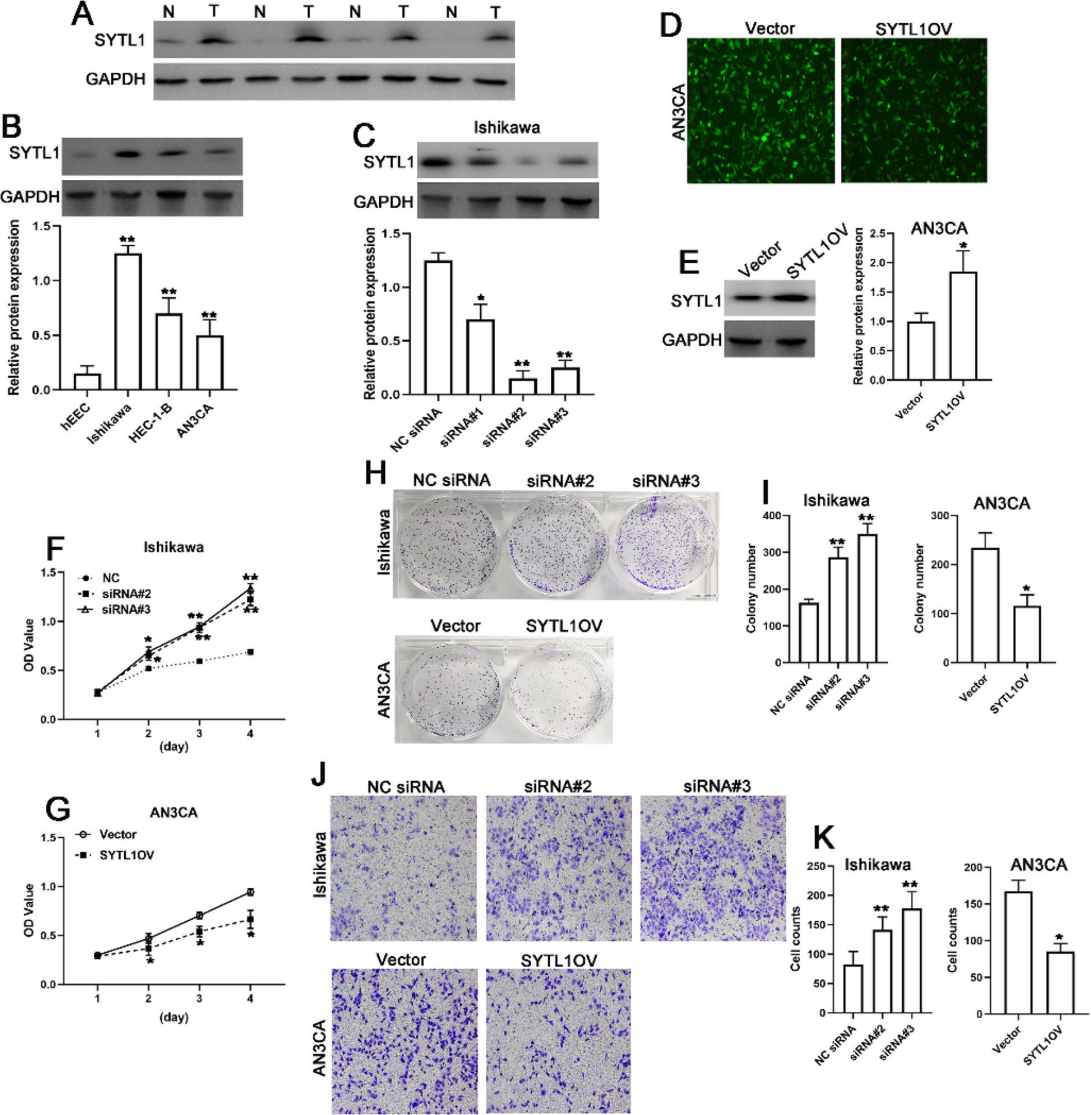
**A**-**B**. The SYTL1 expression profiles in tissues (**A**) and cell lines (**B**). **C**. The protein levels of SYTL1 in Ishikawa cells transfected with SYTL1 siRNA#1, siRNA#2 or siRNA#3. **D**-**E**. Green fluorescence signals (**D**) and western blot (**E**) detecting the efficiency of SYTL1 overexpression in AN3CA cells infected with virus particle. F-G. CCK8 analysis of cell survival of Ishikawa cells (F) or AN3CA (G) cells. H-I. Colony formation analysis of cell proliferation of Ishikawa cells or AN3CA cells. J-K. Transwell assay analysis of cell invasion of Ishikawa cells or AN3CA cells. ***p < 0.001, **p < 0.01

To confirm the function of SYTL1 in EC, we detected the protein levels of SYTL1 in EC patients. Compared with the adjacent non-cancer tissues, the protein levels of SYTL1 in EC tissues were significantly increased (Fig. 7 A). Similarly, SYTL1 protein levels in EC cell lines (Ishikawa, HEC-1-B and AN3CA) were significantly upregulated compared with the hEEC cells in Fig. 7B. These data revealed that SYTL1 was upregulated in UCEC tissues and cell lines.

We synthesized three siRNAs targeting SYTL1 for SYTL1 silencing. In Fig. 7 C, compared with NC siRNA, siRNA#2 and siRNA#3 had better inhibitory efficacy among the three siRNAs in Ishikawa cells. As shown in Fig. 7D, green fluorescent signals were found in LV-GFP (Vector group) and LV-SYTL1-GFP-infected (SYTL1OV group) AN3CA cells, suggesting that AN3CA cells were successfully infected with virus particles. The protein expression levels of SYTL1 group were significantly upregulated in SYTL1OV group displayed in Fig. 7E. The CCK-8 assay revealed that the silencing of SYTL1 significantly increased the survival of Ishikawa cells compared to the respective NC group, however the overexpression of SYTL1 significantly blocked the AN3CA cells survival (Fig. 7 F and 7G). Similarly, the knockdown of SYTL1 in Ishikawa cells induced the colony formation, and SYTL1 overexpression blocked the AN3CA cells colony formation (Fig. 7 H and 7I). In Fig. 7 J and 7 K, the transwell assay results showed that the blockage of SYTL1 enhanced the Ishikawa cell invasion and SYTL1 overexpression in AN3CA cells suppressed the cell invasion. These data indicated that SYTL1 is involved in cell proliferation and cell invasion in EC.

### Differentially expressed genes and functional enrichment analysis

**Fig. 8:**
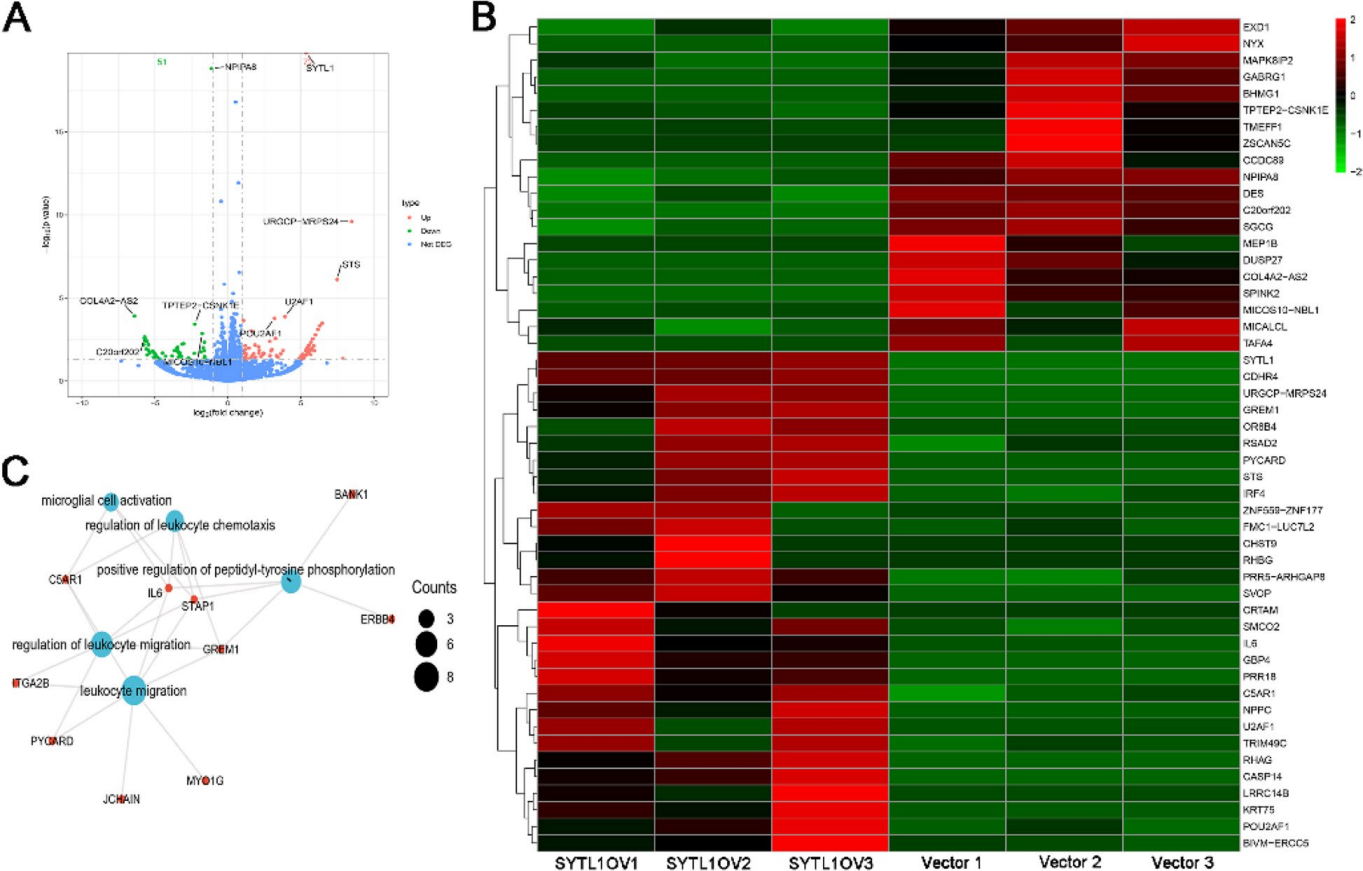
**A**. The volcano plot of differentially expressed genes across LV-GFP infected versus LV-SYTL1-GFP infected AN3CA cells. Red or Green circles referred to statistically significant differentially expressed genes with Log2FC no more than 1.0 and *p* < = 0.05 between two groups. **B**. The heatmap of top 50 upregulated genes and 50 downregulated genes. **C**. GO and KEGG enrichment analysis of the upregulated differentially expressed genes in LV-SYTL1-GFP-infected- versus LV-GFP transfected AN3CA cells. Vector, LV-GFP infected AN3CA cells. SYTL1OV, LV-SYTL1-GFP infected AN3CA cells

To explore the mechanism by which SYTL1 was involved in EC progression, we performed next high-throughput RNA-sequencing in SYTL1 overexpressing AN3CA cells to identify differentially expressed genes and biological functions associated with SYTL1. A total of 123 differentially expressed genes, 72 upregulated and 51 downregulated, were identified in SYTL1 overexpressed cells compared with Vector control cells shown in volcano plot (Fig. 8 A, detailed in Supplement data). The top 50 significantly up-regulated and down-regulated differentially expressed genes were shown in heatmap (Fig. 8B). KEGG functional enrichment analysis revealed that the main biological pathways implicated in upregulated differentially expressed genes were leukocyte migration, regulation of leukocyte migration, positive regulation of peptidyl-tyrosine phosphorylation, regulation of leukocyte chemotaxis and microglial cell activation.

## Discussion

EC is the most common gynecological cancer. Five-year survival rates of EC patients are strictly related to stage at diagnosis. Metabolomics, as an emerging “omics”, has been a promising test for a non-invasive diagnosis of EC. Some reports showed that serum metabolites were able to predict the presence of EC progression and recurrence and pathological characteristics [16]. Additionally, the increasing mortality is closely related with a poorly reproducible histological risk stratification. The TCGA molecular groups and the classic clinicopathological factors (such as myometrial invasion, histotype or lymph vascular space invasion) have been incorporated into a novel risk stratification model of EC by the European Society of Gynaecological Oncology (ESGO), the European Society for Radiotherapy and Oncology (ESTRO) and the European Society of Pathology (ESP) [17, 18]. New reports revealed the distribution and prognostic value of the TCGA groups, and proposed the improvement in the molecular-based risk stratification [19]. Preoperative molecular classification is very useful to guide clinical management by providing earlier and more reliable prognostic information. However, the management of MMR-deficient and no specific molecular profile carcinomas is still difficult. Therefore, the development of novel molecular biomarkers for EC diagnosis and prognosis assessment represents one of the greatest challenges.

The proteins containing the C2 domain play important roles mainly in membrane fusion, exocytosis, cellular trafficking, cell signaling and cancers [20]. SYTL1is a member of tandem C2 domains containing proteins and it is previously revealed to induce the secretion of prostate-specific antigen by prostate cells [5] and secretion of azurophilic granules by granulocytes [7]. Furthermore, SYTL1 regulates exocytosis of secretory lososomes by CTL [4] and blocks amylase secretion by pancreatic acinar cells [8]. Through a literature search, we found that SYTL1 expression and its potential diagnostic and prognostic impact on UCEC has not been explored. In our work, we comprehensively analyzed the diagnostic and prognostic value of SYTL1 in UCEC based on TCGA data and the molecular features of protein phosphorylation, genetic alteration and DNA methylation. In our study, we found that SYTL1 is highly expressed in EC tissues than in adjacent normal tissues and SYTL1 expression is negatively associated with the SYTL1 DNA methylation. In addition, we also found that increased SYTL1 expression is correlated with various clinicopathologic characteristics and the number of immune cell infiltration. Taken together, this study indicated the potential role of SYTL1 during EC pathogenesis and revealed the value as a potential diagnostic and prognostic biomarker in EC.

To data, no research has reported a role of SYTL1 in EC. In the present study, we evaluated the SYTL1 expression profile on the basis of various databases including TCGA and HPA. According to the analysis, the expression of SYTL1 on mRNA and protein levels in UCEC tissues was higher than that in normal tissues, and the results were confirmed by in vitro experiments. Furthermore, ROC results indicated the potential diagnostic value of SYTL1 in EC (AUC = 0.801). Logistic regression analysis revealed that the expression level of SYTL1 was closely associated with the clinical parameters including age, clinical stage, histological type, and histologic grade. Univariate and multivariate analysis further showed that SYTL1 expression was an independent factor for UCEC patient’s prognosis. KM plotter analysis revealed that patients with elevated SYTL1 had longer OS, as well as clinical stage (Stage I &Stage II), histological grade (G3), and tumor invasion. Therefore, our study provided new evidence that SYTL1might be a diagnostic and prognostic biomarker for good survival in UCEC. These findings reveal the role of SYTL1 from a new perspective and enrich the research of SYTL1.

SYTL1is a member of the synaptotagmin-like protein family of secretory factors and it is mainly regulates secretion and exocytosis. Previous study indicated that SYTL1 is transcriptionally activated by nuclear factor-κB and up-regulated by tumor necrosis factor αin prostate cancer cell lines [11]. In addition, SYTL1 could bind to the plasma membrane via interacting withPIP3 [12]. Importantly, PIP3 play a central role in critical cellular processes, such as cell proliferation and survival during tumor development [13]. These evidences revealed that SYTL1 might be involved in cancer progression. Our functional enrichment analysis found that SYTL1 was closely correlated with ribosome, Hippo signaling pathway, Cell cycle and RNA transport. Additionally, our in vitro experiments indicated that ectopic expression of SYTL1 affected cell proliferation and invasion in Ishikawa and AN3CA cells, which verified the results of bioinformatics analysis. In this study, KEGG functional analysis by the next high-throughput RNA-sequencing indicated that the upregulated differentially expressed genes were mainly involved in leukocyte migration, regulation of leukocyte migration, and regulation of leukocyte chemotaxis. Leukocytes migration are critical for an anti-tumor immune response [21]. These results showed that an increase on SYTL1 expression might activate leukocyte migration and chemotaxis to suppress the progression of EC.

Previous study reported that AKT phosphorylated SYTL1 at serine 241, and the phosphorylation of SYTL1 may regulate vesicular trafficking by limiting the availability of SYTL1 to the membrane-bound Rab27A [22]. However, little research reported the relationship between SYTL1 phosphorylation and tumorgenesis. In our research, we found at least 4 predicted phosphorylation sites in EC and a high expression level of SYTL1 phosphorylation level at the S392 locus in the primary tumors compared with normal tissues. These data revealed that the phosphorylation of SYLT1 at S392 might be involved in EC development. Several findings related to the regulation of exocytosis by protein phosphorylation have been accomplished in synaptic membrane-trafficking studies. Additional experiments are required to further evaluate the potential role of SYTL1 phosphorylation at the S392 site and to explore the related mechanism.

DNA methylation of promoter regions is an important mechanism during carcinogenesis [23]. Aberrant DNA methylation has been reported to be an early step during EC development [24]. Via UALCAN portal and TCGA Wanderer database, we found that the difference in promoter methylation level was statistically significant between tumor and normal tissues. We also found that the methyaltion level of nine CpG sites significantly correlated with survival probability. Therefore, it is possible that decreased methylation of SYTL1 DNA could be used as a factor in assessing prognostic confidence.

Tumor microenvironment, consists of immune cells, mesenchymal cells, endothelial cells and inflammatory mediators, has a significant impact on tumor development, chemoresistance and clinical outcomes [25]. Previous study showed that tumor microenvironment in EC has a significant prognostic value and plays a role in resistance to treatment [26, 27]. NK cells, DCs, macrophages and neutrophils-and adaptive B cells and T cells, including cytotoxic T lymphocyte (CD8 + T or CTL) cells, helper (CD4 + T) cells and NT K cells-immune cells are involved in EC development [28, 29]. In our study, we found that SYTL1 expression had a significantly correlation with the number of various immune cells, including NK CD56bright cells,Th17, Neutrophils, Cytotoxic cells, NK CD56dim cells,pDC, iDC, T cells, Treg cells, Macrophages, T helper, Tcm, Tgd, Th2 and aDCs. The strongest correlation was observed between the number of NK CD56bright cells (positive correlation),Macrophages (negative correlation) and SYTL1 expression. It is also reported that RhoA-GAP GMIP associates with the secretory factor JFC1 and regulates actin remodeling and exocytosis in innate immune cells [9]. NK cells are important factors during the pathogenesis of inflammatory and autoimmune disease [30]. NK CD56bright cells mainly mediate antitumor response as a potential cancer immunotherapy [31]. Macrophages are critical drivers for cancer initiation and progression, and the infiltration of macrophages in tumors closely correlates with poor prognosis [32]. Our study indicated that SYTL1 may have a potential influence on EC immunity by regulating immune cell infiltration, ultimately affecting the patient prognosis.

Taken together, our study indicated the statistical correlation between SYTL1 expression and clinical prognosis, DNA methylation and immune cell infiltration in EC. Although these data provided evidence that SYTL1 could be used as a promising diagnostic and prognosticbiomarker in EC, it has some limitations. Firstly, more experiments need to be carried out to explore the specific function of SYTL1 in tumor microenvironment. Secondly, although we found that ectopic expression of SYTL1 modulates cell proliferation and invasion, the detailed mechanism remains unknown. Thus, further studies are needed to be performed to investigate the pathways of action of SYTL1 in EC.

## Materials and methods

### Data sources

The pan-cancer RNA-seq data were downloaded from the publicly available TCGA official website (https://genomecancer.ucsc.edu/).Level 3 HTSeq-fragments per kilobase per million (FPKM) of Uterine Corpus Endometrial Carcinoma (UCEC) samples including 552 tumor tissues and 35 adjacent normal tissues were obtained from TCGA website for further analysis. The normalized microarray GSE17025 obtained from the GEO database contained 12 normal endometrium samples and 91 EC samples [33].

### Gene expression analysis

The expression of SYTL1 between tumor tissues (Cervical squamous cell carcinoma and endocervical adenocarcinoma (CESC),Ovarian cancer (OV), UCEC) and normal tissues were analyzed in GEPIA database (Gene Expression Profiling Interactive analysis, http://gepia.cancer-pku.cn/).ENCORI (The Encyclopedia of RNA Interactomes, http://starbase.sysu.edu.cn/) [14] and the UALCAN portal (http://ualcan.path.uab.edu/) [34] databases provide the differential expression analysis of SYTL1 in UCEC. The Human Protein Atlas (https://www.proteinatlas.org/) provides a broad amount of proteomic and transcriptome information of district human samples. Protein immunohistochemistry of SYTL1 in normal human tissues and UCEC tissues were downloaded from HPA.

### Survival and clinicopathological correlation analysis

UCEC patients were divided into 2 groups (high- and low-risk groups) according to the median expression of SYTL1 in further study. The relationship between SYTL1 expression and clinicopathological characteristics was delineated using the R-package “ggplot2”. According to the high and low-risk value, a survival curve was delineated by using the R-package “survminer” and “survival”. The clinicopathological correlation analysis was mainly performed using the R-pakcage “limma” and “ggpubr”. The association between SYTL1 expression and survival was verified by Kaplan-Meier plotter tool (http://kmplot.com/analysis/) [35].

### LinkedOmics database analysis

The LinkedOmics database (http://www.linkedomics.org/login.php) contains multi-omics data from all 32 TCGA Cancer types and 10 CPTAC cancer cohorts [36].We screened the differentially expressed genes linked to SYTL1in the TCGA UCEC cohort by using the “LinkFinder” module. Gene Oncology (GO) and Kyoto Encyclopedia of Gene and Genomes (KEGG) pathways were analyzed by using “Function” module.

### SYTL1 DNA methylation analysis

TCGA analysis in UALCAN portal was used to analyze the SYTL1 promoter methylation levels in UCEC. TCGA Wanderer, which offers level 3 TCGA data for methylation arrays (450k Infinium chip), was employed to analyze gene expression and DNA methylation profiles from TCGA. MethSurv (https://biit.cs.ut.ee/methsurv/) web tool was used to examine the correlation between individual probes with methylation changes and survival probability [37].

### Immune infiltration analysis using ssGSEA and TISIDB

The spearman correlation between SYTL1 and 24 types of immune cells were evaluated by suing the GSVA package in R. Furthermore, TISIDB, an online web portal for tumor and immune system interaction was used to analyze the distribution of SYTL1 expression across immune and molecular subtypes in “Subtype” module [38].

### Samples collection

A total of 4 pairs of EC and adjacent tissues were collected from patients who underwent surgical resection at Qilu Hospital of Shandong University (Qingdao) from May 2018 to October 2019. EC tissues were collected according to the inclusion criteria: ① complete pathological and clinical data, ②no hormone therapy, intrauterine device usage, chemotherapy or radiotherapy for at least 6 months prior to surgery. EC tissues were excluded according to the criteria: ① patients with malignant tumors of other systems, ② patients with metastatic cancers from other reproductive systems, ③ patients with a history of other hospital treatment. All specimens were evaluated by at least two pathologists according to the World Health Organization guidelines. This work was approved by the Ethics Committees of Qilu Hospital of Shandong University (Qingdao) ([Approval no. (KYLL(2016-KS-173). Permission from all patients was obtained prior to the surgery.

### Cell culture and transfection

Human endometrial epithelial cells (hEEC, #354984), EC cell lines Ishikawa (#338359) and AN3CA (#339020) were obtained from BeNa Culture Collection (Xinyan, Henan, China). EC cell lines HEC-1-B (ZQ0364) was purchased from Shanghai Zhong Qiao Xin Zhou Biotechnology Co.,Ltd. (Shanghai, China). Cells were cultured at 37°C with 5% CO2. Ishikawa cells were incubated with Dulbecco’s modified eagle medium (DMEM) supplement with 10% fetal bovine serum (FBS). AN3CA and HEC-1-B cells were cultured with Eagle’s Minimal Essential Medium (EMEM, Gibco, Carlsbad, CA, United States) containing 10% FBS. For SYTL1 knockdown, Ishikawa cells were transfected with three small interfering RNAs targeting SYTL1 (siRNA#1, siRNA#2 and siRNA#3) and negative control (NC) siRNA. The siRNA sequences were listed as follows: NC siRNA sense: 5’-uucuccgaacgugucacgutt-3’, antisense: 5’-acgugacacguucggagaatt-3’. SYTL1 siRNA#1 sense: 5’-gcugcugugaaagagaaggaatt-3’, antisense: 5’-uuccuucucuuucacagcagctt-3’; SYTL1 siRNA#2 sense: 5’-cccuguguucaaucacaccautt-3’, antisense: 5’-auggugugauugaacacagggtt-3’; SYTL1 siRNA#3 sense: 5’-ccucccggauaagcagagcaatt-3’, antisense: 5’-uugcucugcuuauccgggaggtt-3’. For overexpression of SYTL1, AN3CA cells were infected with LV-GFP (Vector) or LV-SYTL1-GFP (SYTL1OV) (GENECHEM, Shanghai, China) according to the manufacturer’s protocol.

### Western blot

Proteins were extracted from tissues by using RIPA buffer (Sigma-Aldrich; Merck KGaA). Proteins were separated by 10% sodium dodecyl sulfate-polyacrylamide electrophoresis and transferred onto polyvinylidene difluoride membrane (Roche, Basel, Switzerland). The membranes were incubated with mouse monoclonal antibodies against SYTL1 (1:1000 dilution; #sc-365,933, Santa Cruz Biotechnology, Inc.) and against GAPDH (1:1000 dilution, #ab59164, Abcam) overnight at 4 °C. After being washed with TBST, the membranes were incubated with rabbit anti-mouse IgG H&L (HRP) (1:5000 dillution; #ab6728, Abcam) at 37 °C for 1 h. The membranes were visualized using an enhanced chemiluminescence system (ImageQuant LAS4000) by the normalization to GAPDH. The band density was determined by relative densitometry using ImageJ Software version 1.50 (National Institutes of Health).

### Cell counting kit-8 (CCK-8) assay

Ishikawa or AN3CA cells were seeded into 96-well plate at the density of 3,000 cells/well. After 1, 2, 3 and 4 days, 10 µL of CCK-8 reagent was added into each well and incubated at 37 °C for 1 h. The optical density value was measured at the wave length of 450 nm by using a microplate reader.

### Colony formation

Ishikawa and AN3CA cells were seeded into a 6-well plate. After 14 d, cells were fixed with methanol for 15 min and stained with 0.1% crystal violet for 30 min.

### Cell invasion assay

Invasion assay was conducted by using the 24-well transwell chambers with 8 μm proe size (COSTAR, USA). The inserted top side of the chambers containing 150 µL of the serum-free medium was inoculated with 3 × 10^4^ cells, whereas 600 µL of the medium with 10% FBS was added to the lower chamber. After 24 h, the cells invaded to the bottom of the membrane were fixed with 4% paraformaldehyde for 30 min and stained with 0.1% crystal violet for 30 min. The stained cells were photographed and counted under a microscope using a 100× magnification.

### High-throughput RNA-sequencing

AN3CA cells were infected with LV-GFP or LV-SYTL1-GFP lentiviral particles. After 48 h, total RNA was isolated by using TRIzol. Quality control of the total RNA samples were quantified using a NanoDrop ND-1000 instrument and qualified using agarose gel electrophoresis. One to two µg total RNA was used for the preparation of the sequencing library according to the following steps: ① mRNA was purified by oligo-dT magnetic beads; ② RNA-seq library was prepared after First Strand cDNA synthesis, Second Strand cDNA synthesis, End Repair, Ligate adapters and PCR amplification. ③ The completed libraries were qualified by Agilent 2100 Bioanalyzer. ④ FastQ data were obtained by high-throughput sequencing for both ends (2 × 150 bp) on Illumina HiSeq instrument.

### Statistical analysis

Univariate and multivariate logistic regression analysis were performed to calculate the associate between SYTL1 expression and clinicopathological characteristics using Cox proportional hazard models. The correlation between DNA methylation probes and SYTL1 expression was tested using the Spearman (r) correlation method. All statistical analysis was performed with R statistical software (version 3.6.3) and SPSS software (version 24.0). A *P* value less than 0.05 is considered statistically significant.

## Supplementary Information

**Additional file 1: Figure S1. **SYTL1 protein in S392 locus was higher in UCEC. A. The putativephosphorylation sites of SYTL1 were analyzed using ProsphoNET database. B and C. Theexpression level of SYTL1 phosphoprotein (S216 and S392) between normal tissues and ECwas analyzed via the UALCAN. **Figure S2. **The mutation features of SYTL1 for the TCGA tumors were analyzed usingthe cBioPortal tool. A and B. The alteraion frequency with mutation type in human cancersand mutation sites in UCEC. C-F. The potential correlation between mutation status andoverall, disease-free, progression-free and disease-specific survival of UCEC was assessed.

## Data Availability

The datasets generated and analyzed are mainly available from TCGA, GEO, HPA, UALCAN, Wanderer, MethSurv, STRING, GEPIA2 and cBioportal that provide free online tools and resources. Some datasets used and/or analyzed during the current study are available from the corresponding author on reasonable request.

## Abbreviations

SYTL1: synaptotagmin-like protein 1
UCEC: Uterine Corpus Endometrial Carcinoma
CESC: Cervical squamous cell carcinoma and endocervical adenocarcinoma
OV: Ovarian cancer
PIP3: phosphatidylinositol 3,4,5-trisphosphate
FPKM: fragments per kilobase per million
TCGA: The Cancer Genome Atlas
HPA: The Human Protein Atlas
GO: Gene Ontology
CPTAC: Clinical proteomic tumor analysis consortium
STRING: Search Tool for the Retrieval of Interacting Genes/Proteins
GSVA: gene set variation analysis
ENCORI: The Encyclopedia of RNA Interactomes
OS: overall survival
GEPIA: Gene Expression Profiling Interactive Analysis
